# Predictors of life satisfaction in elders living at home in the Czech Republic

**DOI:** 10.1371/journal.pone.0283772

**Published:** 2023-03-30

**Authors:** Radka Bužgová, Radka Kozáková, Katka Bobčíková, Renáta Zeleníková

**Affiliations:** Department of Nursing and Midwifery, Faculty of Medicine, University of Ostrava, Ostrava, Czech Republic; Government Institute of Medical Sciences, INDIA

## Abstract

**Objectives:**

The aim of this cross-sectional study was to determine the life satisfaction of older people living in a home environment and to find out what predictors influence it.

**Methods:**

The research involved 1,121 older people 60 years and above from the Moravian-Silesian region who live in a home environment. The short form of the Life Satisfaction Index for the Thirds Age (LSITA-SF12) was used to assess life satisfaction. The Geriatric depression scale (GDS-15), the Geriatric Anxiety Inventory Scale (GAI), The Sense of Coherence Scale (SOC-13), and the Rosenberg Self-Esteem Scale (RSES) were used to evaluate related factors. In addition, age, gender, marital status, education, social support, and subjective health assessment were evaluated.

**Results:**

The overall life satisfaction score was found to be 36.34 (s = 8.66). The satisfaction of older people was classified into four grades: high satisfaction (15.2%), moderate satisfaction (60.8%), moderate dissatisfaction (23.4%), and high dissatisfaction (0.6%). The predictors of the longevity of the lives of older people were confirmed, both health factors (subjective health assessment, anxiety, and depression [Model 1: R = 0.642; R^2^ = 0.412; p<0.000]) and psychosocial factors (quality of life, self-esteem, sense of coherence, age, and social support [Model 2: R = 0.716; R^2^ = 0.513; p<0.000]).

**Conclusion:**

In implementing policy measures, these areas should be emphasized. The availability of educational and psychosocial activities (e.g. reminiscence therapy, music therapy, group cognitive behavioural therapy, cognitive rehabilitation) within the community care of the older people and university of third age is appropriate to increase the life satisfaction of the older people. An initial depression screening is also required as part of preventive medical examinations to ensure early diagnosis and treatment of depression.

## Introduction

By 2050, it is estimated that one in four people will be over 60 years old, with the fastest relative increase in the over-80 age group [1]. The problems that old age bring and the adaptation to it can influence the assessment of the quality of life of the elderly [2]. With an ageing population and an increase in the number of older people in the population, the assessment of life satisfaction, quality of life, and mental health in old age is a subject of increased interest. Perceptions of life satisfaction, happiness, and quality of life are always very individual. Many physical and mental losses can cause personal discomfort, but coping with these difficulties can help elderly people experience satisfaction with life even in a changed situation [3, 4].

Several authors have defined life satisfaction. For example, Diener et al. [5] defines life satisfaction as cognitive assessment of the overall quality of life. The concept of life satisfaction can also be defined as a feeling of satisfaction with how a person has lived their life up to now and how they evaluate satisfaction with their current life [6]. Most definitions of life satisfaction emphasize the subjectivity of assessment. People are satisfied and happy when they feel that way or when they say that they feel that way (subjectively evaluating that they are satisfied with life and talking about it). Our research was based on this fact. We assume that people have life satisfaction if they feel that way themselves regardless of other circumstances [7]. In the process of assessing life satisfaction, individuals consider the personal priorities of their life, consider it as a whole, weigh the good with the bad, and define it as more or less satisfactory [8].

Contentment with life in old age is considered a success for older people and is associated with healthy ageing and full adaptation to old age [9]. It is also taken as an indicator of better quality of life [10] and successful ageing [11, 12]. It also benefits from better physical health [13] and a lower risk of depression [14]. If an older person is not satisfied with his or her life, he or she may begin to experience despair. Sone et al. [15] found that subjectively perceived life satisfaction can protect the brain from accelerated ageing.

Furthermore, research suggests that the satisfaction of life of older people is influenced by a number of factors that merit investigation. They are related to the level of education, finances, self-assessment of health, satisfaction with social support, and cohabitation in the common household [16]. The influence of psychosocial factors is also confirmed by other research [17–19]. In particular, decreases in social connections and physical functioning in the elderly population are the most frequently reported negative aspects of ageing, thus reducing life satisfaction [20]. Puvill et al. [21] found that mental health has a greater influence on life satisfaction than physical health. The satisfaction of life for older people may also be related to physical activity. The systematic review of Bai et al. [22] provides limited evidence showing the effect of physical activity (fast and moderate walking) on life satisfaction.

Scientific research should aim to identify the key factors that influence the satisfaction of life and quality of life of older people [23]. This can be beneficial for identifying appropriate preventive and policy measures that can have a positive effect on improving the life satisfaction of elderly people, and thus their personal well-being, quality of life, and contribution to maintaining their health.

The aim of this cross-sectional study was to identify the satisfaction of life of older people living in a community environment and to identify what predictors influence it. Based on the abovementioned research, we assumed that life satisfaction may be influenced by both health conditions (physical and psychological) and psychosocial factors.

## Materials and methods

### Sample

The research involved 1,121 older people from the Moravian-Silesian Region who live in a home environment. Older people who did not agree to cooperate and lived in institutional care were not included in the investigation. The criteria for inclusion in the research sample were being aged 60 or older, being cognitively intact (no diagnosed dementia, ability to sign an informed consent), having the ability to understand the Czech language. The exclusion criteria were as follows: disapproval of signing informed consent, mental problems with difficulties understanding and completing the questionnaire.

Older people were approached in all districts of the Moravian-Silesian Region through more than 10 organizations (e.g., senior clubs, community centres), through libraries, and also through the Centre for Prevention and Promotion of Healthy Aging…. The questionnaires were distributed to older people in both printed and electronic form from September 2021 to March 2022. According to data from the Czech Statistical Office in 2021, there are approximately 236,000 persons over 65 years of age living in the Moravian-Silesian Region. Our sample represented 0.5% of these older people.

### Data collection

To assess life satisfaction, we used the LSITA-SF12 (the Life Satisfaction Index for the Thirds Age—Short form) questionnaire, which contains 12 items with options on the 6-point Likert scale: Strongly Disagree (6), Disagree (5), Somewhat Disagree (4), Somewhat Agree (4), Agree (2), and Strongly Agree (1). For Items 2, 4, 5, and 6, the responses are reversed. The total scores for the 12 items to establish the Life Satisfaction score (from 12–72). Higher scores mean lower life satisfaction [22]. We classified overall scores based on quartiles into four grades: high satisfaction (12–27 points), moderate satisfaction (28–42 points), moderate dissatisfaction (43–57 points), and high dissatisfaction (58–72 points). The development of the LSITA scale achieved excellent reliability with Cronbach α = 0.93 [24]. The internal consistency evaluated using Cronbach’s alpha in our research sample was found to be satisfactory at 0.88.

Anxiety and depression were measured in the psychological health assessment. Depression was measured using the Short Form of Geriatric Depression Scale (GDS-15) consisting of 15 questions (response: yes/no). Scores of 0–4 are considered normal; 5–8 indicate mild depression; 9–11 indicate moderate depression; and 12–15 indicate severe depression. The GDS-15demonstrated good reliability and validity [25]. Fountoulakis et al. [26] published a high internal consistency of GDS-15 with Cronbach’s alpha 0.94. In our research also satisfactory reliability of the GDS-15 scale was found (Cronbach’s α = 0.85).

Anxiety was assessed using the Geriatric Anxiety Inventory Scale (GAI), which consists of 20 “agree/disagree” items designed to assess typical common anxiety symptoms. The total score ranges from 0–20, and higher scores indicate more anxiety. A good internal consistency was found in the target group (Cronbach’s α = 0.91 in healthy older people and 0.93 in psychogeriatric patients), high test-retest validity and correlation with other anxiety detection methods [27].

The Sense of Coherence Scale (SOC-13) and the Rosenberg Self-Esteem Scale (RSES) were also evaluated. The SOC-13 scale consists of 13 items that comprise three components: SOC_C—comprehensibility (5 items), SOC_MA—manageability (4 items), and SOC_ME—meaningfulness (4 items). Respondents indicate agreement or disagreement on a seven-category semantic differential scale with two anchoring responses tailored to the content of each item. The total score can range from 13–91, and a higher score indicates a higher SOC [28]. Data from several studies reporting the reliability of the SOC-13 scale have generally found acceptable reliability indicators, which, measured with Cronbach’s alpha, range from 0.70 to 0.93 for the SOC-13 [29]. Excellent reliability was found in our research (Cronbach’s α = 0.92).

The RSES scale [30] is a 10-item Likert-type scale, with items answered on a four-point scale: from strongly agree to strongly disagree. The total scores range from 0–30. Higher scores mean higher self-scores. A score of less than 15 means low self-esteem. Mayordomo et al. [31] confirmed the validity and reliability (Cronbach’s α = 0.73) of the RSES scale in elderly population. Very good reliability of the RSES scale (Cronbach’s α = 0.81) was also found in our elderly population.

Quality of life (QoL) was assessed using the Older People Quality of life brief—OPQOL_brief questionnaire [32]. The scale consists of 13 statements. The items scores are totalled to provide a total OPQoL-brief score ranging from 13 to 65, with higher scores indicating better QoL [32].

Age, sex, marital status, cohabitation, employment, and social support were assessed from social factors. Social support was evaluated only from the subjective point of view of the person surveyed by the question: “Do you have the impression that you have people close by who will give you help and support if you need it?” on a scale of 1 (yes, always) to 10 (no, never).

Physical health was assessed by the number of illnesses regularly seen by the doctor and by a subjective health assessment, which was evaluated by one item: “How would you rate your health status?” with the possible answers: 1. Bad (I need assistance), 2. Rather bad (my health significantly restricts me in my daily activities), 3. Rather good (my health does not significantly restrict me in my daily activities) and 4. Good (I feel fully healthy and do not perceive restrictions in my daily activities).

### Ethical aspects

The study was in accordance with the provisions of the Declaration of Helsinki and was approved by the ethics committees of the Faculty of Medicine, University of Ostrava (no. 14/2020). All subjects gave their written informed consent to inclusion before participating in the study.

### Data analysis

The SPSS statistical program, v. 24.0 was used for data analysis. Data were evaluated using descriptive statistics (absolute and relative frequency, mean, standard deviation). Differences between groups were evaluated using the Kruskal–Wallis test and the independent Wilcoxon test. The correlation between the parameters was determined using Spearman’s correlation coefficient. Nonparametric tests were used due to abnormal data distribution (Kolmogorov–Smirnov test, p<0.001). Multivariate regression analysis was performed using the enter method, employing all variables that showed significant association (p<0.05) in primary analysis. A p-value < 0.05 was considered statistically significant.

## Results

The socio-demographic characteristics of the population are shown in Table 1. Elderly patients also reported how many diseases they were regularly treated for. The average number of diseases per older person was found to be 2.6. The most frequent older people reported being treated for cardiovascular (62.8%) and musculoskeletal (49.1%), sensory (31.9%), and urological diseases (19.4%).

**Table 1:** Socio-demographic and health characteristics of sample (n = 1 121).

Characteristics			Characteristics	N	%
**Age**			**Employment**		
Mean (SD)	72.7	6.5	Full time	49	4.3
Min (max)	60	89	Part-time job	124	10.9
**Social support** (scale 1–10)	2.4	1.9	No	948	84.8
**Illnesses**	**Mean**	**SD**	**Living with**		
Number of disease/1 person	2.6	1.7	Alone	454	40.1
**Gender**	**N**	**%**	Spouse	540	47.7
Man	302	26.9	Children	88	7.7
Women	819	73.1	Another	39	3.3
**Marital status**			**Subjective health assessment**		
Single	35	3.0	Poor	41	3.6
Married	542	47.9	Fair	180	16.0
Divorced	183	16.1	Good	648	57.9
Widow	361	31.9	Very good	252	22.5

**Fig 1:**
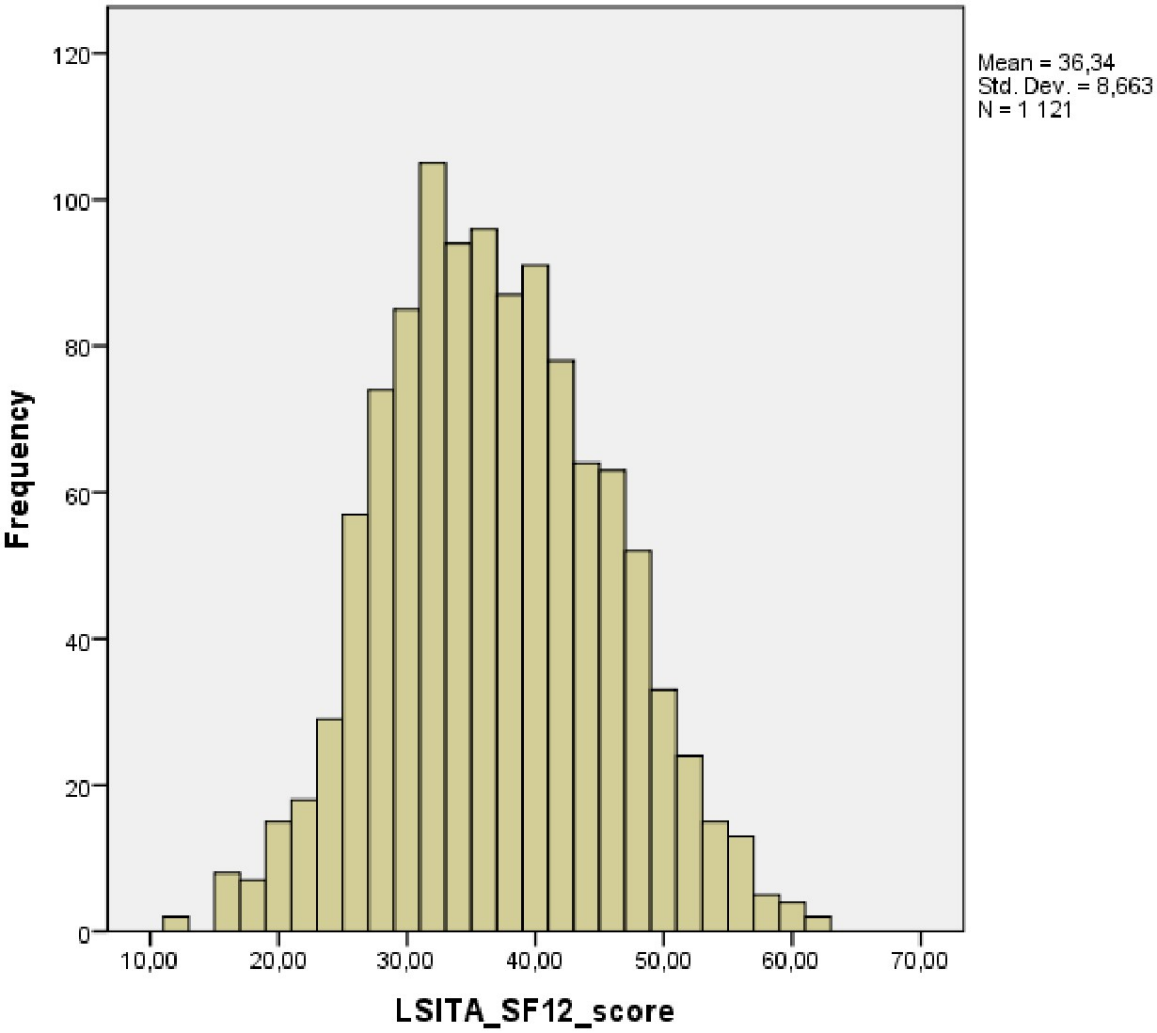
Contribution of LSITA_SF12 scale.

The total life satisfaction score was 36.34 (CI = 35.83–36.85), standard deviation = 8.66, median = 36.00, min-max = 12–62, skewness = 0.144, kurtosis = -0.264, see Fig 1. Older people reported the highest moderate satisfaction (n = 682; 60.8%). A total of 170 (15.2%) older people reported high satisfaction. On the contrary, 262 (23.4%) older people reported moderate dissatisfaction, and only 7 (0.6%) older people reported high dissatisfaction.

First, we evaluated the difference in life satisfaction scores for the individual groups of older people. Table 2 shows that a statistically significant difference was found in all monitored parameters. Women reported higher life satisfaction, then employed older people, older people living with someone in the same household, married, under 75 years of age, subjectively assessing their health as good or very good, with no signs of depression (GDS<5) and higher self-esteem scores (RSES>15).

**Table 2:** Evaluation of differences in life satisfaction according to selected factors.

	mean	Sd	p		mean^a^	sd	p
**Gender**				**Age**			
Man	37.45	8.78	**0.016**	≥75 (n = 429)	38.60	8.62	**0.000**
Women	35.99	8.61		<75 (n = 692)	34.94	8.40	
**Employment**				**Subjective health**			
Yes	32.79	7.73	**0.000**	Very good/good	34.76	7.98	**0.000**
No	36.95	8.74		Fair/poor	43.04	8.21	
**Living**				**GDS**			
Alone	37.39	8.83	**0.001**	0–4 (n = 802)	33.25	7.21	**0.000**
With	35.67	8.51		5 a vice (n = 319)	44.61	7.00	
**Marital status**				**Religion**			
Single	37.79	9.85	**0.000**	Yes	35.38	8.68	**0.028**
Married	35.34	8.29		No	36.63	8.59	
Divorced	35.60	8.55		**RSES**			
Widow	38.14	8.90		0–15 (n = 120)	46.49	6.70	**0.000**
				16–30 (n = 1001)	35.64	8.33	

^a^Higher LSITA-SF score means worse life satisfaction

A correlation was found between higher life satisfaction ratings and lower rates of depression (r = 0.688; p<0.001) and anxiety (r = 0.450; p<0.001). A correlation was also found between higher life satisfaction ratings and better cohesion ratings for SOC (r = -0.532; p<0.001), SOC_C (r = -0.545; p<0.001), SOC_MA (r = -0.357; p<0.001), SOC_ME (r = -0.560; p<0.001), RSES self-esteem (r = -0.519; p<0.001), quality of life (r = 0.592; p<0.001), social support (r = 0.292; p<0.001), subjective health (r = -0.416; p<0.001), and lower age (r = 0.223; p<0.001).

Tables 3 and 4 show predictors of life satisfaction for older people. Two models that included health and psychosocial factors were tested. Health factors included both mental and physical health. Both models were confirmed to be statistically significant. The predictors explained 41.2% of the variation in health factors and 51.3% of the variation in psychosocial health factors. The predictors of better life satisfaction are both health factors (better subjective health assessments, lower depression and anxiety) and psychosocial factors (better quality of life assessments, self-esteem, sense of coherence, lower age, and better social support assessments).

**Table 3:** Multiple regression analysis with the health factors as independent variables and the life satisfaction as dependent variables (model 1).

Health factors R = 0.642; R^2^ = 0.412; P<0.000	B	SE	95% CI for B		β	t	P
			low	up			
**Subjective health**	-0.1997	0.362	-2.707	-1.286	-0.156	-5,513	**0.000**
**GDS (depression)**	1.444	0.099	1.250	1.639	0.518	14,545	**0.000**
**GDA (anxiety)**	0.119	0.055	0.011	0.227	0.073	2,169	**0.030**
**Constant**	36.764	1.252	34.307	39.220	—	29,376	**0.000**

**Table 4:** Multiple regression analysis with the psychosocial factors as independent variables and the life satisfaction as dependent variables (model 2).

Psychosocial factors R = 0.716; R^2^ = 0.513 P<0.000	B	SE	95% CI for B		β	t	P
			Low	up			
**QoL**	0.470	0.036	0.399	0.540	0.381	13,038	**0.000**
**RSES**	-0.560	0.073	-0.702	-0.418	-0.234	-7.717	**0.000**
**SOC-13**	-0.165	0.026	-0.216	-0.115	-0.199	-6.398	**0.000**
**Age**	0.097	0.033	0.032	0.162	0.073	2.927	**0.004**
**Social support**	0.332	0.109	0.117	0.546	0.081	3.033	**0.002**
**Constant**	37.952	3.211	31.651	44.254	—	11.821	**0.000**

## Discussion

The aim of our research was to find out what factors influence the life satisfaction of the elderly. Based on previous research, we assumed that life satisfaction might be influenced by psychosocial factors, as well as by the assessment of mental and physical health. Life satisfaction in the elderly is an important topic because it can play a crucial role for successful ageing [9, 11, 12], the quality of life of the elderly [10], their physical health [13], the reduction of depression [14], as well as hopelessness [33]. Jose, George and Dante [34] found that older people with high levels of life satisfaction experience relatively low levels of anxiety about death and vice versa. These are the reasons why policy measures should contribute to influencing factors that contribute to better life satisfaction.

In our research, it was confirmed that the predictor of life satisfaction is the subjective assessment of both physical and mental health, anxiety, and depression (model 1). Differences were also found when comparing between groups. Older people who had at least mild depression reported worse life satisfaction. Similarly, older people who evaluated their physical health as fair or poor reported lower life satisfaction. In previous research, the existence of chronic diseases [10, 35, 36], physical limitations in everyday life [35], depression [35, 37], and anxiety [18] were found to be the main determinants of life satisfaction. Older people with better health status showed greater satisfaction. Puvill et al. [21] demonstrate the greater impact of mental health compared to physical health. Depression is considered the most common mental health problem among older people and the most common psychiatric disorder [38] that negatively affects the life of older people [39]. Geriatric depression increases the risk of other diseases, cognitive impairment, and premature death or suicide, and it increases the cost of treatment [40]. Of the psychological factors that influence life satisfaction, it may not only be anxiety and depression, but also fear [41]. Especially in this day and age, when older people are threatened by fear of COVID-19, isolation, or war, it can become a significant predictor. However, this factor has not been investigated in our investigation.

The second model tested confirmed the influence of psychosocial factors such as quality of life, self-esteem, sense of coherence, age, and social support on the evaluation of life satisfaction. Social support in our research was tested only by the subjective view of the person surveyed by one question, which represents a certain limit of research. On the other hand, it is the subjective perception of social support that can indicate satisfaction or dissatisfaction with social support, regardless of the number of people and contacts the person has.

Sex, education, employment, cohabitation, and religion were also found in the difference testing. The improved life satisfaction was reported in our research by women, as well as older people who are in the labour force, live with someone in the same household, are married/divorced, are religious, and rate their self-esteem better.

Social support [10, 18, 36], living in a marriage, or possibly cohabiting with another person in the same household [7, 10, 42], can make a significant contribution to the life satisfaction of the elderly, as evidenced by previous research. Widows reported lower life satisfaction. Among widows, older people with more children were better off [42]. In addition to family relationships, it can also be the level of other social interactions that influence how older people assess their life satisfaction [35].

Pan et al. [37] compared the influence of social factors and physical functioning. Social functioning measured by the number of people living together, social support, and the sense of loneliness contributed more to life satisfaction than physical functioning (self-perceived health, ADL, IADL, number of chronic illnesses).

Age was also found to be a predictor of life satisfaction in our research. Older people under 75 years of age showed greater satisfaction. In the research of Tavares et al. [35], life satisfaction increased with age from the age group 70–74 years. Pan et al. [37] found that older age and lower levels of education were associated with higher levels of life satisfaction.

Some research has focused on other factors where the positive influence of hope has been demonstrated [2, 42, 43], such as spiritual well-being [2] and sufficient satisfaction of physical and psychological needs [17].

In our research, we tested self-esteem and sense of coherence, which have not been tested as predictors of life satisfaction in previous research. The author who coined the term “sense of coherence” is Antonovsky [28], who talks about understanding the image of the world as a meaningful whole and considers this factor to be a *salutor*—i.e., a positive factor improving health despite adverse environmental influences. Zielinska-Wieczkowska et al. [23] state that the sense of coherence (SOC) is one of the most important factors determining life satisfaction and the ability to cope with difficult situations accompanying old age. Meaningfulness of life is then an important motivating element, stimulating one to understand the outside world, in a difficult situation that can be typical for the elderly. A person with strong SOC will be able to cope with many difficult life situations, which will contribute to their life satisfaction. A fundamental result of our research is confirmation of the knowledge that sense of coherence and self-esteem positively influence the assessment of life satisfaction.

Further research should focus on effective preventive strategies that support mental health, including reducing symptoms of anxiety and depression and increasing the quality of life or sense of coherence of the elderly [44]. Salvi [38] recommends using psychosocial interventions such as cognitive behavioural therapy, reminiscence therapy, problem-solving therapy, or cognitive therapy (mindfulness-based cognitive therapy) for the prevention and treatment of depression [39] investigated the effectiveness of interventions that promote a positive understanding of ageing and create a positive attitude toward old age among the elderly. They found that positive psychological interventions have positive effects on mental well-being and the reduction of depression and anxiety among older people, improving life satisfaction, hope, and positive emotions. In addition to psychosocial interventions, some studies have also shown a positive effect of educational programs within third-age universities [45–48]. Older people can gain increased life satisfaction, self-esteem, and self-confidence through third-age university programmes. At the same time, they have more social interactions that can reduce their loneliness. Further research should focus on the effectiveness of these measures.

### Policy implications or recommendations

As part of policy measures aimed at preventing the effects of an ageing society and the related promotion of healthy ageing, it is appropriate to focus on improving mental health (reducing anxiety and depression), reducing loneliness, promoting a sense of coherence and self-esteem among the elderly. This activity can be carried out by community centres in non-profit organisations working with the elderly, or by third role universities (Universities of Third Age, Centres for the Prevention and Promotion of Healthy Ageing in Educational and Research Centres). A government agency can help to support the funding of these activities.

Educational activities, reminiscence therapy, music therapy, group cognitive behavioural therapy, art therapy, and cognitive rehabilitation should also be offered in these community centres for older people living in the community. These activation activities are now being realised, especially in institutional care for older people. Educational activities within third-age universities and other educational institutions for the elderly should focus on the prevention of the most frequently occurring diseases in old age such as cardiovascular and musculoskeletal disease. The advantage of third-age universities is that they can provide education in the field of disease prevention while increasing the social contacts of older people, their participation in society, self-esteem and the sense of coherence. Therefore, they can have a positive effect not only on physical health, but also on mental health of older people [49].

Appropriate measures should be taken to prevent, detect early, and diagnose depression. General practitioners should play a crucial role and may also include depression screening as part of preventive screening. We recommend the use of the GDS-15 scale, which allows for rapid screening of depressive symptomatology. The GDS-15 scale has been widely used for the screening of depression and was valid for measuring mild and major depression [50]. Subsequently, the general practitioner may send selected older people for a more detailed psychiatric evaluation for diagnosis and suggestion of appropriate treatment for depression. Here, too, the above-mentioned community centres can play an important role and may also perform initial detection using the GDS-15 scale.

## Conclusion

In our research, predictors of the life satisfaction of older people living in a community have been confirmed, both health factors (subjective health assessment, anxiety, and depression) and psychosocial factors (quality of life, self-esteem, sense of coherence, age, and social support). In implementing policy measures, these areas should be emphasized. The availability of educational and psychosocial activities in community care for older people is appropriate, as well as the motivation of older people to participate in these activities.

## Supporting information

S1 Data (SAV)
